# Detecting non-natural language artifacts for de-noising bug reports

**DOI:** 10.1007/s10515-022-00350-0

**Published:** 2022-08-24

**Authors:** Thomas Hirsch, Birgit Hofer

**Affiliations:** Institute of Software Technology, Graz University of Technology, Inffeldgasse 16b, 8010 Graz, Austria

**Keywords:** NLP, Bug reports, Issue tickets, Data cleaning, Artifact removal, De-noising

## Abstract

Textual documents produced in the software engineering process are a popular target for natural language processing (NLP) and information retrieval (IR) approaches. However, issue tickets often contain artifacts such as code snippets, log outputs and stack traces. These artifacts not only inflate the issue ticket sizes, but also can this noise constitute a real problem for some NLP approaches, and therefore has to be removed in the pre-processing of some approaches. In this paper, we present a machine learning based approach to classify textual content into natural language and non-natural language artifacts at line level. We show how data from GitHub issue trackers can be used for automated training set generation, and present a custom preprocessing approach for the task of artifact removal. The training sets are automatically created from Markdown annotated issue tickets and project documentation files. We use these generated training sets to train a Markdown agnostic model that is able to classify un-annotated content. We evaluate our approach on issue tickets from projects written in C++, Java, JavaScript, PHP, and Python. Our approach achieves ROC-AUC scores between 0.92 and 0.96 for language-specific models. A multi-language model trained on the issue tickets of all languages achieves ROC-AUC scores between 0.92 and 0.95. The provided models are intended to be used as noise reduction pre-processing steps for NLP and IR approaches working on issue tickets.

## Introduction

Textual documents produced during the software development process are increasingly popular targets for natural language processing (NLP) and information retrieval (IR) approaches. Specifically issue tickets have drawn the attention of researchers and practitioners. Such techniques are applied to issue tickets to categorize the impact and root causes of bugs (Zhou et al. 2021), to classify bugs according to the Orthogonal Defect Classification (ODC) scheme (Thung et al. 2012), to assign programmers to bug reports (Mani et al. 2019; Devaiya et al. 2021), to locate the source code that needs to be changed to fix a bug (Zhou et al. 2012; Saha et al. 2013; Ye et al. 2016), to label the severity of a bug (Kumar et al. 2021; Kukkar et al. 2019), to prioritize bugs (Ortu et al. 2016), to detect duplicates (Kukkar et al. 2020), to distinguish bug reports from other issues (Chawla and Singh 2015), and to find security related bug reports (Goseva-Popstojanova and Tyo 2018).

Unfortunately, issue tickets are often cluttered with non-natural language artifacts such as code snippets, stack traces, log outputs, and configuration files. Such artifacts inflate the size of issue tickets^1^ and pose a problem for some of the above mentioned tasks. Some approaches require that all artifacts are removed from the text, e.g. analysis of developers’ personalities (Calefato et al. 2019) and language identification (Jauhiainen et al. 2017). In other approaches, the artifacts should not be eliminated, but processed separately. For example, Bacchelli et al. (2012) investigated the content of development emails and argued that source code, stack traces, and other artifacts should not be part of the same bag of words as natural language. In our approach on multi-class root cause classification based on bug reports (Hirsch and Hofer 2022a), we have observed that artifacts have an impact on specific classes’ performance, either being beneficial or detrimental to its performance.

Since artifacts also decrease readability for humans, issue trackers usually provide formatting mechanisms, such as Markdown, that allow authors to format their issue tickets accordingly. Parsing issue tickets along these formatting rules and markup languages is probably the simplest and easiest form of artifact detection. Unfortunately, not all ticket authors use these formatting tools properly.^2^ Therefore, formatting alone is not a viable option for reliable artifact detection.

Researchers developed numerous techniques for identifying and parsing such non-natural language artifacts. A popular technique are regular expressions tailored to the underlying dataset (Tan et al. 2014; Ray et al. 2014; Soltani et al. 2020). Although labor intensive, this approach works reasonably well for homogeneous datasets containing a rather small set of different types of artifacts, e.g., data originating from only a small number of software projects that are composed in the same programming language and targeting the same execution platform.

However, such sets of regular expressions lack in transferability to other, or new data, and have to be adapted accordingly. To the best of our knowledge, there is no standard corpus of regular expressions for this task. Manual identification of artifact patterns in new data combined with the adaption or expansion of the set of regular expressions is time-consuming, as pointed out by Mäntylä et al. (2018). Manually created rule sets do not scale to larger, heterogeneous datasets due to the size and number of required regular expressions necessary to account for different logging frameworks, code style guidelines, built systems, configuration file formats, underlying OSs, and IDEs. These scalability and portability issues led researchers to the application of machine learning (ML) techniques (Mäntylä et al. 2018; Bacchelli et al. 2012). While ML approaches circumvent the manual creation of rules, they introduce the need for manually annotated training sets.

In this work, we propose a supervised machine learning approach combined with an automated training set creation process implemented in Python. Our approach does not require extensive knowledge about the artifacts that are supposed to be removed, while providing good classification performance at a low computational cost once trained. Our automated training set creation process locates instances in the dataset that can be labeled using heuristics based on GitHub Markdown. The resulting fractions of the original data sets are used to train models with the purpose of generalizing the classification problem again to the whole range of input data. Our models can classify inputs that are not Markdown annotated. In contrast to general purpose NLP pipelines, we perform custom, task specific, tokenization. We evaluate our models on manually annotated validation sets randomly sampled from our original datasets.

This paper is based on previous work (Hirsch and Hofer 2021) presented at the 2nd International Workshop on Software Engineering Automation: A Natural Language Perspective (NLP-SEA 2021) co-hosted with ASE. While the workshop paper focused on the comparison with existing work (Mäntylä et al. 2018), we now focus on the portability of the approach. For this purpose, we extend our evaluation from Java projects to projects written in four popular programming languages, namely C++, JavaScript, PHP, and Python. We answer the following research questions in this journal paper:

- **RQ1: Do different underlying programming languages affect the performance of our artifact detection approach?** While we have designed our approach to be language independent, we have not empirically evaluated this aspect in the workshop paper. However, it is important to empirically evaluate this aspect, because syntax and stack traces differ for different programming languages and it is unclear whether the used features are well suited for other programming languages. For example, curly brackets and semicolons are heavily used in C++ and Java, but these symbols are rarely used in Python.
- **RQ2: Are artifact detection models trained on one programming language transferable to other programming languages?** Here, we investigate if a model trained on bug reports of projects written in one programming language can be used to correctly detect artifacts originating from other programming languages.
- **RQ3: What is the performance of a multi-language model for artifact detection?** In practice, projects are often written in several programming languages and therefore bug reports might contain code snippets and stack traces of several programming languages. A multi-language model might be particularly useful in such scenarios.

Since we already compared our approach to NLoN (Mäntylä et al. 2018) in the workshop paper (Hirsch and Hofer 2021), the comparison of our approach and NLoN is not part of this journal paper and we refer the interested reader to the workshop paper.

The remainder of this paper is structured as follows: Sect. 2 discusses the related work. Section 3 describes the problem of distinguishing natural and non-natural language parts and we define where we draw the line between natural language and artifact. Section 4 explains the automatic creation of the training set, the features used in the ML approach, the preprocessing steps, and the used machine learning models. Section 5 deals with the setup and the results of the empirical evaluation. Section 6 concludes the paper.

## Related work

Natural Language Processing (NLP) and Information Retrieval (IR) approaches on textual documents from software development processes often require identification of non-natural language portions or identification of specific types of artifacts. Regular expressions and island parsers (Bettenburg et al. 2008; Bacchelli et al. 2011; Rigby and Robillard 2013) are currently amongst the most popular methods to perform such a separation. While these methods can be implemented in a reasonable amount of time for a homogenous set of issue tickets concerning a single language and similar context, they do not scale well for a large number of issue tickets from heterogeneous sources, spanning multiple domains, companies, and programming languages (Calefato et al. 2019).

InfoZilla (Bettenburg et al. 2008) extracts structural information such as stack traces, source code, patches, and enumerations from bug reports using regular expressions, island parsing and heuristics. The approach was evaluated by manually classifying 800 bug reports from the Eclipse issue tracking system.

Bacchelli et al. (2011) used island parsing to extract structured data from natural language documents. They evaluated their approach on the mailing lists of three large open-source Java projects. In later work, they proposed a supervised ML approach to classify the content of emails line-by-line into natural language, junk, code, patch and stack trace. To train and test the classifier, they manually classified the content of nearly 1500 emails from four software systems (Bacchelli et al. 2012).

Rigby and Robillard (2013) developed an island parsing-based tool called Automated Code element Extractor (ACE) that automatically extracts code elements such as packages, types, and methods. They empirically evaluated ACE on StackOverflow posts that used one of the tags HttpClient, Hibernate, or Android.

Ponzanelli et al. (2015) used island parsing to identify Java code, stack traces, XML/HTML elements and JSON fragments in natural language text. They provide a parsed dataset, named Stack Overflow Ready Made Data (StORMeD), that contains heterogeneous abstract syntax trees for the identified non-natural language fragments.

Calefato et al. (2019) reported on their experiences when using regular expressions to remove code snippets from email text: they found this approach does not scale well enough—in particular when several programming languages are used. This highlights the need for more generic approaches for artifact detection such as machine learning.

Ye et al. (2017) use a semi-supervised machine learning approach to detect API mentions in text written on social platforms. They evaluated their approach on Stack Overflow posts to identify API mentions of three well-known Python libraries.

While the above described approaches aim at identifying certain types of artifacts (e.g. stack traces Bettenburg et al. 2008; Bacchelli et al. 2011; Ponzanelli et al. 2015, JSON fragments Ponzanelli et al. 2015, or API mentions Ye et al. 2017), we aim at separating natural language from non-natural language artifacts in general, indifferent to the specific type of the artifact. Due to the differing goals and scopes, we cannot quantitatively compare our approach to the above solutions.

The work that is closest to ours is the Natural Language or Not (NLoN) Package (Mäntylä et al. 2018). This R package classifies text lines into text or artifact by using eleven language features and character tri-grams. The approach is trained and evaluated on three data sources (i.e. comments from the Mozilla issue tracker, chat entries from Kubernetes, and emails from Apache Lucene’s mailing list archive), each containing 2000 data samples that were manually labeled as natural text or artifact. The major differences between NLoN and our approach are the explicit language features used in NLoN, and the training set creation process: While NLoN relies on a manually labeled training set, we automatically generate the training sets. NLoN’s hard coded features makes it applicable to C++, Java and similar languages, but would require adaptions to be used for Python. Further, we implemented our approach in Python, as Python has surpassed R in popularity.^3^

## Problem definition

This paper proposes an automated approach to distinguish natural language text portions from non-natural language artifacts on a line-by-line basis. Table 1 illustrates this distinction on an excerpt from Bazel issue 3906.^4^

**Table 1:**
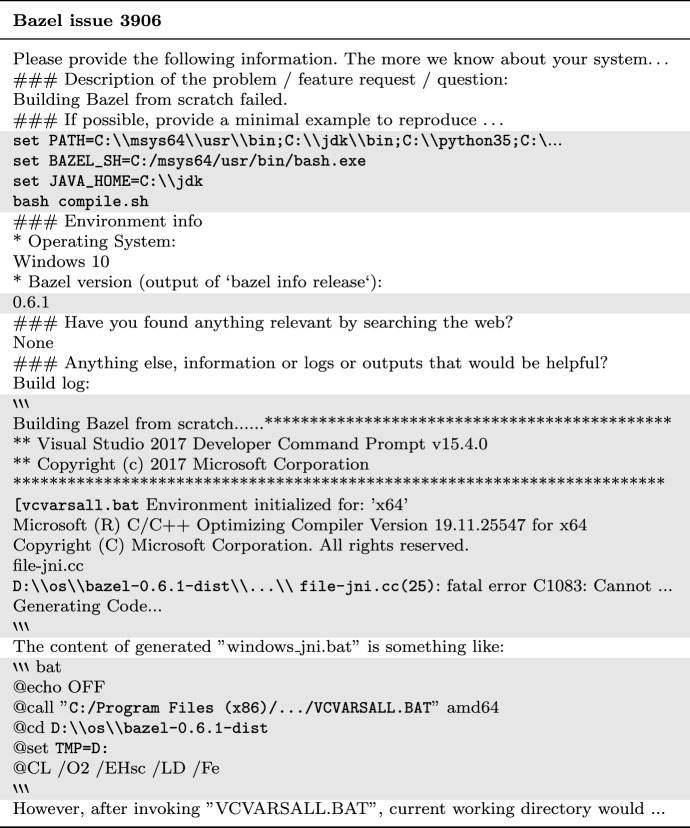
Natural language and artifacts (shaded in gray) categorization for an excerpt of Bazel issue 3906

Our intuition tells us that the line between natural language or non-natural language should be a clear cut. However, closer investigation reveals the complexity of this problem and gray areas where the two categories overlap. Examples of such border cases are code comments and issue ticket templates: Comments contained in code snippets are natural language texts. However, they may not have been authored by the issue reporter. Issue ticket templates consist of headers, questions, and other texts (see header lines starting with ### in Table 1 for an example). While they are natural language, they are again not written by the issue reporter and are to be considered automatically generated text. Migration from other issue tracking systems often introduces generated text portions. They are also natural language, but their origin is artificial. Such text portions are highly repetitive and may add very little value to the downstream NLP or IR task. Product and version numbers are another example of text that is difficult to distinguish on a line by line basis. While short identifiers like Windows 10 are often seen as human-written, verbose and detailed version identifiers often seem to be copy-pasted. In particular when the version numbers of several programs, apps, or environment settings are listed^5^, it is more likely that the information was copy-pasted. As far as we are aware, there exists no formal definition, established guideline, or agreement within the research community working with textual issue tickets on what is to be considered natural language when dealing with issue tickets.

For this work, we define artifacts and natural language portions of issue tickets as indicated in Table 2. We consider text that was typed by the reporter of the issue ticket as natural language, and content that was originating from an IDE, terminal, or other tool to be an artifact. Automatically generated natural language text of the issue tracking tool, template, or migration processes is considered natural language. Comments in pasted code snippets, elaborate natural language logging messages and error messages are considered artifacts. Further, we consider standalone URLs and Markdown links as artifacts. We treat standalone numbers such as version numbers as artifacts, but the combination of product names and version numbers as natural language.

**Table 2:** Categorization into natural language and artifacts

Natural language	
Text typed by issue reporter	
Issue ticket template text	
Natural language sentence containing variable names	
Natural language sentence containing URLs/Markdown links	
Natural language text migrated from other issue tracking systems	
Product name and version number	

Artifact	
Content originating from IDE, terminal, $\dots$	
Code snippets and code comments	
Error messages, stack traces	
Standalone URLs/Markdown links	
Log output	
Standalone numbers, e.g., version numbers	

Occurrences of non-natural language portions in a natural language sentence are mostly limited to variable names, class names, and short formulas or mathematical equations. Removing such occurrences may render a natural language sentence syntactically and semantically incorrect and unreadable for a human. We therefore consider a line of natural language text interweaved with non-natural language portions as natural language.

We approach the task as a line by line binary classification problem, similar to Mäntylä et al. (2018). While intuitive, as log outputs or code snippets always start on a new line, distinguishing classes based on an isolated line can be challenging due to lack of contextual information. Examples for such cases are code comments, and natural language error messages contained in log outputs.

## Approach

Our approach essentially consists of three steps: (1) our automated training set creation process, described in detail in Sect. 4.1, (2) feature engineering, as discussed in Sect. 4.2, and (3) our preprocessing and supervised machine learning pipeline, presented in Sect. 4.3.

### Automated training set creation

A major advantage of our approach is that manual annotation of a training set is not required. Instead, we rely on Markdown annotated portions of the input dataset to automate the training set creation process. GitHub’s built-in issue tracker offers Markdown^6^ to format issue reports. Given our task at hand, we focus mainly on the following Markdown features: Triple ticks that start and end a code highlighting block, indentation by four spaces signaling a code block, lines that are entirely in quotes, Markdown style links, tables, URLs, and embedded images. Markdown code block highlighting features are extremely well suited for our purpose of building a line by line approach. For example triple tick code blocks have their Markdown annotations signaling begin and end in separate lines. Having no inline markup annotations and formatting rules inside of such code blocks, prevents formatting information leaking into the contained lines, and prevents overfitting on Markdown features.

**Fig. 1:**
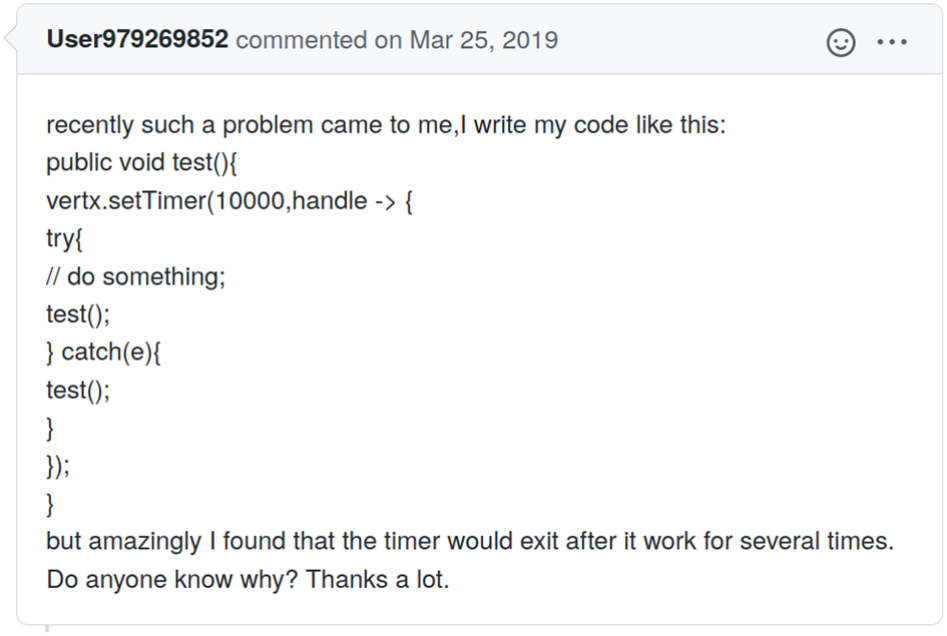
Eclipse-vertx vert.x issue 2887 (https://github.com/eclipse-vertx/vert.x/issues/2887) as an example of an issue ticket that should have used Markdown to highlight code

If all issue reporters would adhere to formatting rules and apply these Markdown features to wrap non-natural language artifacts, the task of artifact removal would be trivial. Unfortunately, this is not the case (see Fig. 1, and Table 1 for examples).^7^

**Fig. 2:**
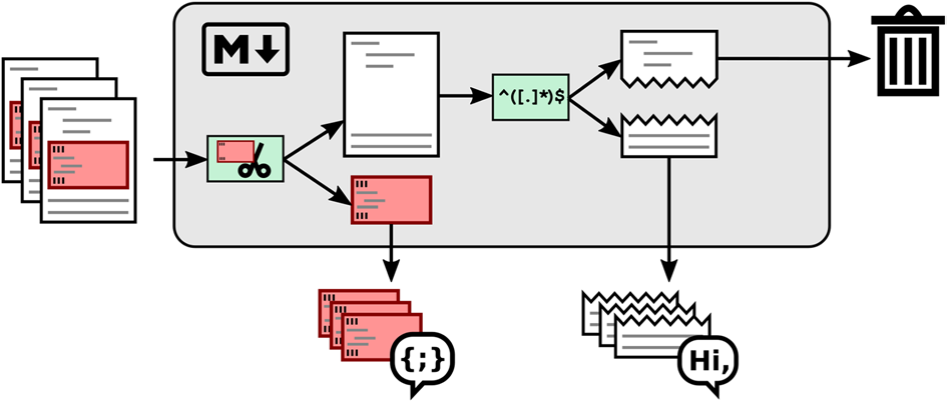
Automated separation of human-written text and artifacts

Due to inconsistencies in Markdown usage, simple Markdown parsing to identify artifacts is insufficient. However, we can leverage the issue tickets that do contain Markdown code highlighting features to create an annotated data set to be used in training ML classifiers. Figure 2 illustrates this process. We use all issue tickets that contain blocks wrapped in triple ticks. Triple tick code blocks have to be deliberately put in place by the issue reporter, showing some awareness to Markdown of the author, in contrast to code block highlighting by indentation. We then split the content of these issue tickets into natural language and non-natural language portions. To do so, we employ a small set of six regular expressions to capture the various Markdown annotated artifacts discussed above. This process is based on the assumption that if reporters utilize Markdown in their issue ticket, they will do so consistently.

However, this assumption does not always hold, and therefore produces supposedly natural language text portions that in fact are artifacts of some kind. To reduce the resulting noise in the natural language portion of the dataset, we apply a set of regular expressions to remove common artifact types. Each line is applied to a sequence of regular expressions, either matching it as an artifact and therefore removing the line, or labeling it natural language if no regular expressions match. The first part of these regular expressions can be easily reused in any context: Two regular expressions remove Unix and Windows style prompts, two regular expressions remove json and xml like content, one regular expression for invalid Markdown quoted text, and one regular expression for hexadecimal numbers. The second part of regular expressions stem from our initial target consisting of Java projects: Four regular expressions specifically aim at Java code, and four regular expressions target logging formats. While our Java specific regular expressions to some degree work to identify C++, PHP, and JavaScript code (e.g. line ending with semicolon or curly bracket), they are unsuitable to identify Python code, and unsuitable to identify logging output from these other languages. We finally use two regular expressions to remove lines whose formatting does not allow to distinguish them via regular expressions (e.g. Markdown block quotes using ‘>’ are used to highlight text^8^, for reply or followup in conversations^9^, and to quote excerpts from the documentation^10^ as well as for code highlighting or error messages^11^). Despite our cleaning efforts, the resulting training sets might be noisy. We will evaluate the amount of noise in the resulting training sets in Sect. 5.3.

**Fig. 3:**
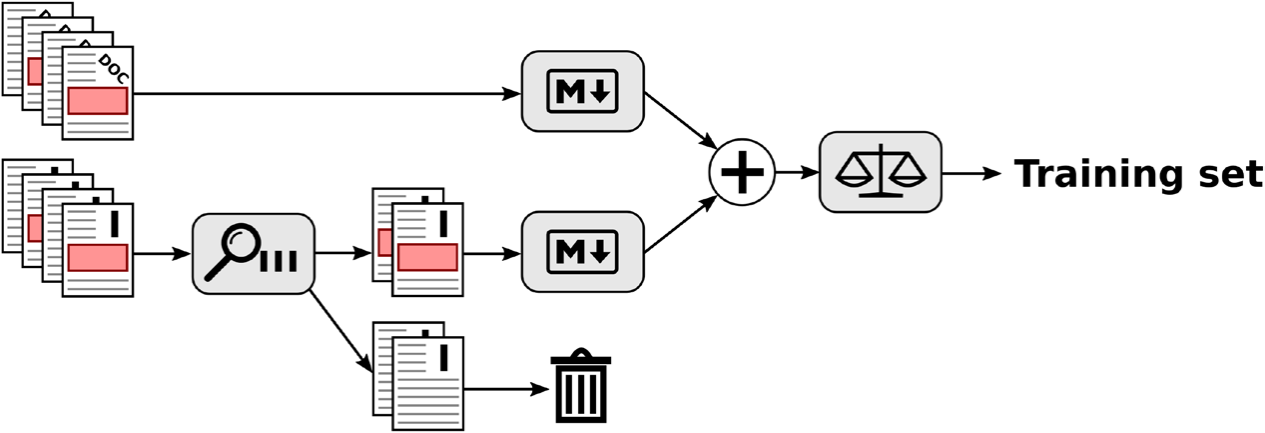
Training set creation process where M$\downarrow$ represents the process of Fig. 2

The process described in Fig. 2 is only applied to those issue tickets which contain Markdown triple ticks. All other issue tickets are discarded and therefore are not part of the training set (see Fig. 3). Besides issue tickets, we use documentation files in Markdown syntax found in the projects’ repositories to augment our training set. The rationale behind this is that project maintainers utilize Markdown consistently in their documentation files. We employ the same approach for the separation of artifacts from natural language as described above for the issue tickets.

The resulting collection of natural language lines and artifact lines is imbalanced. Since the machine learning algorithms used in this work are sensitive to such imbalance, we apply downsampling. We described our sampling strategy in more detail in Sect. 5.3.

### Feature selection

Humans can separate artifacts from natural language without actually reading a text. We therefore attempt to identify the features that enable humans to perform this task so easily.

Formatting and structure in particular help humans to classify text segments very fast. For example, indentation of code snippets provides a very good indicator. Therefore, we will include representations of whitespaces in the feature vectors used by the ML classifier.

A closer look at artifacts further reveals that frequency and position of special characters also carry a significant amount of information for our task. While the most common special characters in English text are ‘,’ and ‘.’, the characters ‘<’, ‘>’, and ‘/’ are probably the most common in XML. For this reason, we tokenize special characters to include them in the feature vectors.

Further, we replace occurrences of camel cased words, underscored words, and numbers with respective tokens, as their type as such, carries more information, than their actual value. The full replacement table can be found in the online appendix; an excerpt of this table is shown in Table 3.

**Table 3:** Excerpt of introduced tokens

Character/regex	Token
*(two whitespaces)*	Jdoublespace
$\backslash$t	Jtabulator
*	Jasterisk
(	Jroundbracketopen
=	Jequals
+	Jplus
{	Jcurlybracketopen
;	Jsemicolon
:	Jcolon
?	Jquestion
([A-Z]?[a-z0-9]+)([A-Z][a-z0-9]*)+	Jcamelcased
([a-zA-Z0-9]+_)+[a-zA-Z0-9]+	Junderscored
[0-9]+	Jnumber
0x[a-f0-9]+	Jhex

Further, the position of a special character contains useful information for the task at hand. Lines of natural language will often end with ‘.’, ‘?’, and ‘!’, while lines of Java code will often end with ‘{’, ‘}’ or ‘;’ but ‘.’ is used to call objects’ methods. A bag of words (unigram) approach is not suitable to encapsulate such position information. Thus, we add tokens that represent the beginning and end of a line, and employ tri-gram vectorization.

### Preprocessing and machine learning approach

We use supervised machine learning classification algorithms and NLP preprocessing steps from an established machine learning library for Python. For detailed background information, we refer the interested reader to Baeza-Yates and Ribeiro-Neto (1999) for an introduction into natural language processing and information retrieval, as well as James et al. (2013) and Bishop (2006) for a more detailed introduction on machine learning.

We use regular expressions and basic string operations to perform the replacements discussed in Sect. 4.2. This step is implemented as a scikit-learn transformer. Doing so enables us to utilize standard tokenization and vectorization functions.

We do not perform stop word removal. Examples for such words in the English language would include articles and pronouns (e.g. “the”, “it”, “we”). Removal of stop words is very commonplace in most NLP and IR applications as they are considered noise with little informational value. However, they provide valuable features for our task, as they are very common in natural language text, and mostly scarce in non-natural language artifacts.

Further, we do not perform case folding, as this also carries some information for the task at hand (e.g. all caps words are more common in artifacts). To encapsulate positional information of the tokens in the feature vectors (as discussed in Sect. 4.2), we vectorize into uni-, bi-, and tri-grams that are combined into a single feature vector using a simple count vectorizer.

We use classic ML models as Support Vector Machines (SVM), Random Forrest Classifier (RFC), Logistic Regression Classifier (LRC), and Multinomial Naive Bayes (MNB), due to their ease of use and little requirements in terms of computational resources for training and prediction. We do not perform hyperparameter tuning, and keep the default values of the classifiers in the used library (MNB: $\textit{alpha}=1.0$, SVM: $C=1.0$, RFC: $\textit{nEstimators}=100$, LRC: $C=1.0$). While automated hyperparameter tuning can offer higher model performance, it comes with a high cost in terms of runtime and increases the risk of overfitting. In this work we chose to use the available time to perform more experiments, e.g. Bootstrap with more repetitions, to increase the sample size of performance scores and confidence in our results.

In a preliminary experiment, the classification performance and capabilities of all classifiers were very similar, but the prediction and training times varied. Given the similarity in classification performances, we chose SVM for the following experiments.

## Results and discussion

We present our research questions in Sect. 5.1, followed by a brief description of the metrics and statistical tests used to evaluate our approach in Sect. 5.2. In Sect. 5.3, we outline the creation process of the datasets and numerically describe the generated training sets, and the manually labeled validation sets in detail. Finally, we present the results of the empirical evaluation in Sect. 5.4 and discuss the threats to validity in Sect. 5.5.

### Research questions

This paper investigates the portability and transferability of our approach onto other programming languages. We address the following three research questions:

**RQ1: Do different underlying programming languages affect the performance of our artifact detection approach?** To answer RQ1, we collect issue tickets from projects written in C++, Java, JavaScript, PHP, and Python. We create manually labeled validation sets for each language. We apply our approach to each language and evaluate it on the corresponding validation set. We plot the learning curves for each language and compute the ROC-AUC values. Further, we discuss performance in terms of training time, prediction time, and resulting model size.

**RQ2: Are artifact detection models trained on one programming language transferable to other programming languages?** To answer RQ2, we train language specific models and evaluate their classification performance on all validation sets. We select a suitable training set size based on our findings in RQ1, and lock it for all experiments to enable comparison.

**RQ3: What is the performance of a multi-language model for artifact detection?** To answer RQ3, we train models on mixed training sets and evaluate their classification performance on each language validation set. We keep the same training set size that we used in RQ2. We create a training set from equal sized portions from each language specific dataset and apply our approach.

**Exclusions:** This journal paper focuses on the portability and transferability of our approach and we do not compare our approach to any baseline or existing approach for the following reasons:

First, there exists only one similar approach, namely NLON (Mäntylä et al. 2018), and a detailed comparison and cross evaluation of our approach to NLON was performed in our previous work (Hirsch and Hofer 2021). The excessive runtime requirements for training NLON on big datasets make it infeasible to evaluate the approach on the bigger datasets used in this work. We therefore refer the interested reader to our previous work (Hirsch and Hofer 2021).

Second, we exclude performance comparison with regex based solutions because there is no standard corpus of regular expressions for the task of artifact removal. The regex sets used by other researchers for the same task are ad-hoc implementations that are either minimalistic, tightly tuned to their specific datasets, or unavailable. The achievable performance of custom regex sets for a given dataset is only limited by time and effort. This voids any meaningful and objective comparison of our models’ performance to existing and custom build regex solutions.

### Evaluation metrics and statistical tests

We use the following metrics and statistical tests in our evaluations:

*True Positives.* True Positives (*TP*) is the number of instances that are correctly identified.

*False Positives.* False Positives *FP*) is the number of instances incorrectly identified as this type.

*False Negatives* False Negatives (*FN*) is the number of instances of a specific type that were not identified.

*True Negatives* True Negatives (*TN*) is the number of instances not belonging to a specific type that were not identified as that type.

*Precision.* The precision indicates the proportion of correctly identified instances based on all instances reported to be of this type, and is computed as follows:

1 $$\begin{aligned} \textit{Precision}=\frac{\textit{TP}}{\textit{TP}+\textit{FP}}. \end{aligned}$$

*Recall.* The recall indicates what proportion of all instances of a type have been classified as such:

2 $$\begin{aligned} \textit{Recall}=\frac{\textit{TP}}{\textit{TP}+\textit{FN}}. \end{aligned}$$

*ROC-AUC.* The Receiver Operating Characteristics (ROC) curve illustrates the ratio of the false positive rate ($\frac{\textit{FP}}{\textit{FP}+\textit{TN}}$) to the true positive rate ($\frac{\textit{TP}}{\textit{TP}+\textit{FN}}$) for different thresholds. The Area Under the Curve (AUC) measures the area of the ROC curve and expresses how good a classifier distinguishes the classes. It has a value between 0 and 1 where a value of 1 means perfect prediction of the classes, while a value below 0.5 indicates that the classification model performs worse than random choice.

*Cohen’s Kappa.* The Cohen’s Kappa coefficient $\kappa$ measures the inter-rater agreement corrected for agreement by chance. It is computed based on the proportion of items where both raters agree ($p_0$), and the proportion of times where agreement is expected by chance ($p_c$) (Cohen 1960) and is computed as follows:

3 $$\begin{aligned} \kappa = \frac{p_0 - p_c}{1 - p_c} \end{aligned}$$

$\kappa$ values between $0.41< \kappa < 0.60$ are considered as moderate, $0.61< \kappa < 0.80$ as substantial, and $0.81< \kappa < 1.00$ as almost perfect agreement (Landis and Koch 1977).

*Student’s T-test and Wilcoxon signed-rank test.* To investigate the significance of differing performance scores of our various models, we perform statistical tests. Whenever the underlying performance scores are normally distributed we report* p*-values from Student’s* T*-test. This is the case for all models’ performance scores on a single language validation set. However, if the underlying data is not normal distributed—as is the case for the mean performance over all languages—we perform Wilcoxon signed-rank test. The null hypothesis assumes that the models have the same mean value and the alternative hypothesis assumes that the mean of the first model is greater than the mean of the second model. If the p-value is smaller than a predefined threshold, the null hypothesis can be rejected. We choose as threshold 0.05.

### Datasets

We create separate datasets for C++, Java, JavaScript, PHP, and Python. We decided to focus on these programming languages, since they are popular in practice (see Stack Overflow 2021 Developer Survey^12^) and research (in particular C++ and Java).

We build our datasets by mining GitHub repositories. We select the 30 most-starred projects for each programming language^13^ excluding educational and non-English projects. The selected projects cover a wide variety of software domains, ranging from server side applications, database applications, ML frameworks, testing frameworks, to mobile applications and games. A complete list of projects can be found in the online appendix. We used the GitHub API to crawl all closed issue tickets regardless of labels (excluding pull requests), as well as the projects’ documentation files.^14^

Table 4 indicates the number of issue tickets crawled for each language and in total. Although we have collected issue tickets from 30 projects for each language, the number of issue tickets per language varies significantly with 41K issue tickets for C++ and 187K issue tickets for JavaScript. From each dataset, we randomly select 250 issue tickets to build the validation set. The remaining issue tickets form the basis for the training sets as described in Sect. 4.1. The removal of data associated with our training set creation approach can be observed in Table 4, with 40-60 % of issues being removed in the initial filtering step.

**Table 4:** Summary of the training and validation sets

	C++	Java	JavaScript	PHP	Python
Number of issues	41 542	131 329	187 340	108 568	159 760
Issues containing MD codeblocks	9 400	46 079	66 319	46 523	95 676
*Training set issue tickets*					
Number of issues	9 343	45 980	66 231	46 422	95 521
Artifact lines	327 615	1 975 090	2 043 327	1 773 366	4 085 192
Natural language lines	82 155	431 281	682 452	529 900	1 716 916
% of natural language lines	20.05 %	17.92 %	25.04 %	23.00 %	29.59 %
*Documentation*					
Number of files	618	683	2 373	511	825
Artifact lines	20 719	27 372	139 937	16 335	37 447
Natural language lines	34 782	36 482	109 988	30 864	51 596
% of natural language lines	62.67 %	57.13 %	44.01 %	65.39 %	57.95 %
*Full training set*					
Lines total	465 271	2 470 225	2 975 704	2 350 465	5 891 151
Artifact lines	348 334	2 002 462	2 183 264	1 789 701	4 122 639
Natural language lines	116 937	467 763	792 440	560 764	1 768 512
% of natural language lines	25.13 %	18.94 %	26.63 %	23.86 %	30.02 %
*Validation set*					
Number of issues	250	250	250	250	250
Artifact lines researcher 1	3 708	4 688	3 240	4 226	8 342
Natural language lines researcher 1	1 708	1 887	1 930	1 964	2 559
Artifact lines researcher 2	3 719	4 672	3 225	4 198	8 427
Natural language researcher 2	1 699	1 906	1 942	1 991	2 556
Cohens Kappa	0.96	0.97	0.96	0.92	0.95
ROC-AUC	0.98	0.99	0.98	0.96	0.97

*Training set.* The training set creation process is illustrated in Fig.3. We remove all issue tickets that do not contain triple tick Markdown code blocks. The remaining issues tickets are subject to the process described in Sect. 4.1, providing a collection of lines labeled either natural language, or artifact. The resulting collections also differ in their size with the Python set as the largest set and the C++ set as the smallest set. Further, all five datasets are imbalanced with natural language as minority class. In order to obtain more natural language data, we include project documentation files. Inclusion of documentation files benefits the training set size most notably for languages with a lower number of available issue tickets (C++) while being a diminishing factor where many issue tickets are available (Python). Documentation files are subjected to the same process as issue tickets as described in Sect. 4.1.

In order to create balanced training sets we perform downsampling. In detail, we randomly sample with replacement *n*/2 lines from each side of a collection to create a balanced training set with size *n*.

**Table 5:** Number of incorrect labeled items from a randomly selected subset of 500 lines of artifacts and 500 lines of natural language from the automatically created training sets

	C++	Java	JavaScript	PHP	Python
Artifacts	6	4	3	2	0
Natural language	21	18	13	11	4

To evaluate the quality of the automatically created training sets, we randomly sampled 500 lines containing artifacts and 500 natural language lines from the collections for each programming language. Researcher 2 manually inspected the samples and marked all lines that contain wrongly labeled data. Table 5 shows the number of incorrectly labeled lines for each sample. The natural language samples contain more noise than the artifact samples. This can be explained by our automated training set creation process: The artifacts side of our collections is sourced from explicitly annotated portions of the input documents, the remainder of those documents are the source for our natural language portion. While it is a rare sight that Markdown code highlighting features are accidentally used for natural language text, the omissions of such Markdown annotations for such artifacts happens far more frequent.

*Validation set.* In order to evaluate our approach, a validation set containing the ground truth is required. Due to the loss occurring in our automated training creation process, a classic test/training split on our training data would produce highly skewed test sets that do not represent the real world. We therefore randomly sample 250 issue tickets from each unfiltered language data set, to be manually inspected and annotated in order to create realistic validation sets. Both authors classified the issue tickets of all five languages. It took each author between 2 and 3.5 hours to classify 250 issue tickets per language. Therefore, a total of 1250 issue tickets, accumulate to approximately 35.000 lines that were manually labeled. In total each researcher spent about 13 hours for labeling all validation sets. The resulting training sets are imbalanced with non-natural language artifacts as the majority class. (see Table 4) For each dataset, we achieved a Cohen’s Kappa interrater agreement between 0.92 and 0.97 (see Table 4), indicating almost perfect agreement.

Table 6 provides details on the researchers’ classifications. About two thirds of the lines were classified as artifacts by both researchers. We manually investigated all lines where we disagreed on the classification. The main reasons for discrepancies are:

- Moments of inadvertence where one researcher incorrectly labeled a line,
- Different opinions on how to classify lines written in other languages than English, e.g., Chinese, and
- Different opinions on how to classify lines containing only a few words and an URL, e.g., ‘- Originally reported by: [mrexodia](http://bitbucket.org/mrexodia)’

**Table 6:** Validation set: agreement and disagreement of the researchers’ classification on 250 randomly selected issue tickets for each language

Researcher 1	Researcher 2	C++	Java	JavaScript	PHP	Python
Artifact	Artifact	67.76 %	70.60 %	61.60 %	66.31 %	75.61 %
Artifact	Natural language	0.70 %	0.70 %	1.05 %	1.94 %	0.92 %
Natural language	Artifact	0.87 %	0.41 %	0.83 %	1.54 %	0.94 %
Natural language	Natural language	30.67 %	28.29 %	36.53 %	30.22 %	22.54 %

*Markdown usage.* In Sect. 4.1, we have stated that only some of the issue reporters use Markdown and that Markdown might not be used consistently. To support this claim, we have manually examined the quality of the issue tickets contained in the validation sets. Researcher 2 manually inspected all issue tickets from the validation sets and classified them into two groups: issue tickets that use Markdown code blocks and issue tickets that do not use Markdown code blocks. For the first group, Researcher 2 inspected if Markdown was consistently and correctly used, i.e., if all code snippets, error messages, log outputs, and stack traces are properly Markdown annotated and if the Markdown annotated code blocks do not contain natural language. Between 3.9 % and 9.2 % did not use Markdown consistently. For the second group, Researcher 2 investigated if the issue tickets contain any code snippets, error messages, log outputs, or stack traces that should have been Markdown annotated. Between 7.4 % and 23.4 % of the issue tickets without Markdown code blocks contain segments that should have been wrapped in a Markdown code block. Table 7 provides the detailed numbers for all validation sets. In general, this table supports our claim that Markdown annotated issue tickets are cleaner than those without annotations.

**Table 7:** Markdown usage in the issue tickets of validation set

	C++	Java	JavaScript	PHP	Python
*Issue tickets using Markdown code blocks*					
Markdown consistently used	79	99	98	103	148
Markdown inconsistently used	7	10	4	8	8
% Markdown inconsistent	8.1 %	9.2 %	3.9 %	7.2 %	5.1 %
*Issue tickets not using Markdown code blocks*					
Markdown not necessary	147	112	137	119	72
Markdown should have been used	17	29	11	20	22
% that should use Markdown	10.4 %	20.6 %	7.4 %	14.4 %	23.4 %

### Empirical results

**RQ1: Do different underlying programming languages affect the performance of our artifact detection approach?** We created balanced training sets of different sizes according to the process described in Sect. 5.3. The training set sizes *n* were 6 250, 12 500, 25 000, 50 000, 100 000, 200 000, 400 000, 800 000, 1 600 000, 3 200 000.^15^ We trained our models on these training sets and evaluated them using the corresponding validation set. This experiment was performed 10 times for each training set size *n*, while resampling the training set for each new iteration.

We performed our experiments on Manjaro Linux 21.1.6 in a Python 3.9 conda environment on an AMD Ryzen 7 Pro 3700U Processor (2.30 GHz, up to 4.00 GHz Max Boost, 4 Cores, 8 Threads, 4 MB Cache) with 16 GB RAM.

**Fig. 4:**
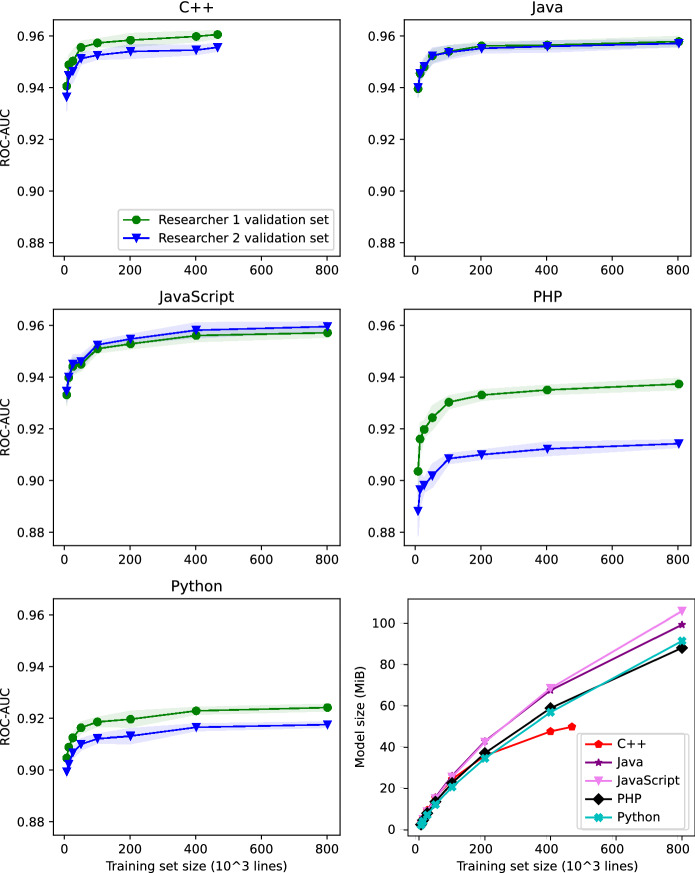
ROC-AUC learning curves and model size for each programming language

Figure 4 shows the results of this experiment. To increase readability, the graphs were truncated at training set size $n=$ 800 000 due to negligible changes in performance beyond this point. The resulting model sizes increase nearly linearly with training size *n*. A similar effect can be observed with the time required for training these models. Both training time and model size are very similar for each underlying programming language.

In order to compare the performance of our approach on different programming languages, the respective models have to be trained with the same amount of data. We chose a training set size of $n=$ 200 000, as to provide reasonably sized models to be used in practice or other research, and we again created training sets for each language according to the process described in Sect. 5.3, and evaluated the trained models on the corresponding validation sets. However, this time we trained and evaluated each programming language model 100 times to provide a bigger sample of performance scores.

**Fig. 5:**
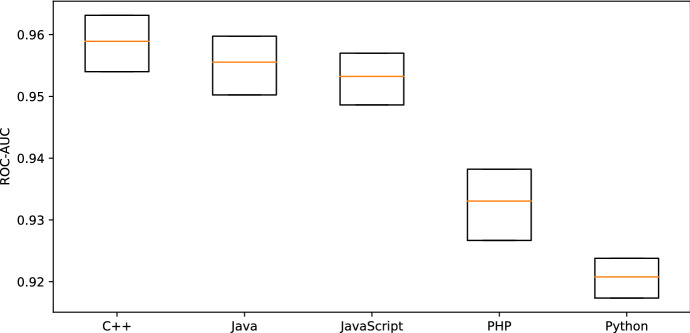
ROC-AUC bootstrap confidence interval ($\alpha =0.95$, 100 repetitions) on Researcher 1 validation sets using training set size $n=$ 200 000 for each programming language

Table 8 shows the mean performance scores resulting from this experiment. Figure 5 shows the bootstrap confidence intervals ($\alpha =0.95$) of our models’ ROC-AUC scores on Researcher 1 validation sets. Our models’ performance depends on the projects’ programming language that source our datasets. Our approach performs best for C++, followed by Java, JavaScript, PHP, and Python in that order. The differences between each of the performance samples is statistically significant. One sided Student’s* T*-test performed on each neighboring language pair shows that C++ performance is better than Java ($p=2*10^{-22}$), and similarly for each consecutive language pair in the above ranking (Java vs. JavaScript $p=6*10^{-12}$, JavaScript vs. PHP $p=4*10^{-117}$, PHP vs. Python $p=2*10^{-79}$).

**Table 8:** Mean performance (100 repetitions) using training set size $n=$ 200 000 for each programming language

	C++	Java	JavaScript	PHP	Python
ROC-AUC reseacher 1	0.96	0.96	0.95	0.93	0.92
ROC-AUC reseacher 2	0.95	0.96	0.96	0.91	0.91
Artifact precision researcher 1	0.98	0.99	0.98	0.99	0.99
Artifact precision researcher 2	0.98	0.99	0.98	0.97	0.98
Artifact recall researcher 1	0.96	0.94	0.95	0.89	0.88
Artifact recall researcher 2	0.95	0.94	0.95	0.88	0.87
Model size (MiB)	35.92	42.92	42.54	37.13	34.58
Training time (s)	110.16	68.57	62.59	90.31	85.77
Prediction time per 5000 lines (s)	0.44	0.35	0.30	0.30	0.31

We observe no correlation of these performance scores to inconsistent usage of Markdown in their corresponding datasets (see Table 7). Further, language features as semicolon line terminators and usage of curly brackets do not seem to be the distinguishing factor in classification performance. PHP syntax features both while significantly performing worse than JavaScript that does not require semicolons as line terminators.

The precision for artifacts of PHP and Python models is comparable with the other languages as shown in Table 8. However, PHP and Python show a significantly lower recall for artifacts. This leads to an increased number of artifacts being wrongly classified as text.

We manually investigated the lines misclassified by our models. The following types of artifacts were often misclassified as natural language text:

- Log outputs that closely resemble natural language, contain, or constitute syntactically correct English sentences,
- Product names and corresponding version numbers, separated by colons (They are often composed by issue reporters and we do not consider them artifacts (see Sect. 3). However, such formatted lines also often occur in log outputs and environment specification files.), and
- URLs and other artifacts that contain a significant amount of English words.

The remaining misclassifications were obvious errors of the models.

**Fig. 6:**
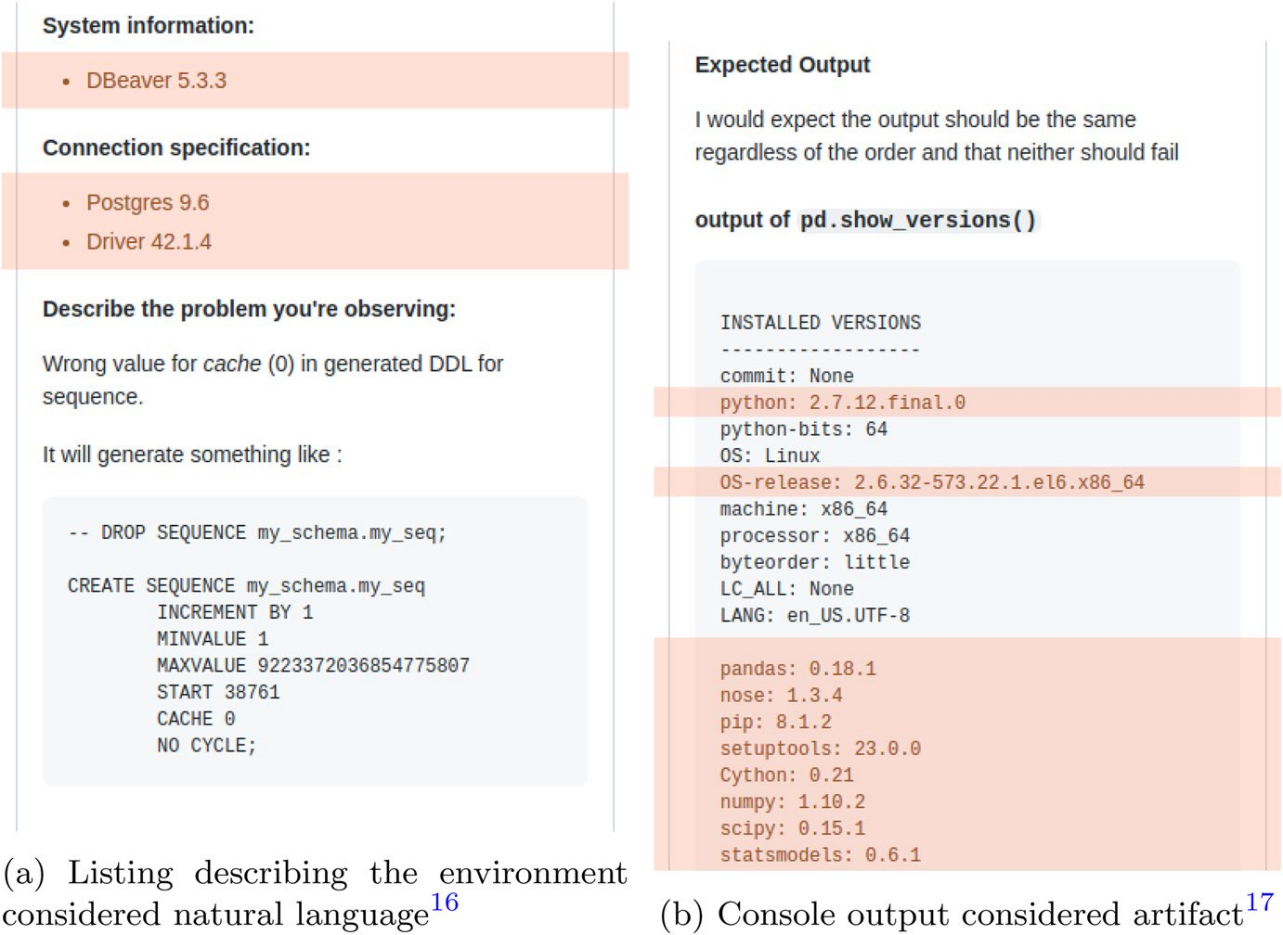
Examples of lines consisting of product and version number to be considered part of natural language, and part of a log-output artifact

Examples of such misclassifications are shown in Table 9. Although rare, obvious mistakes and URLs shown at the bottom of Table 9, seem to occur in all language models. However, the majority of misclassifications on our PHP validation sets stem from log outputs that closely resemble or constitute English sentences. Further, product and version number combinations make up the absolute majority of misclassifications on our Python validation sets and also often occur in our PHP validation sets. In Sect. 3 we formulated that we consider product/version number combinations as natural language, as such segments occurring in Java issue tickets are mostly short, human written, and surrounded by natural language. However, as the comparison of the bug reports^16^,^17^ in Fig. 6 highlights, Python and PHP issue tickets contain such product/version number combinations often as parts of console outputs and log outputs that we considered non-natural language artifacts. Manual annotation of our validation sets was performed on an issue ticket level, providing the annotators the necessary context information to decide if a specific line is part of such a console output artifact or not. This contextual information is not available to our line-by-line classifier models.

Both problems are not solvable with our current approach given our definition of non-natural-language artifacts in Sect. 3. However, depending on the downstream task’s requirements, this definition and our approach could be modified to consider all occurrences of product/version number combinations as non-natural artifacts.

**Table 9:** Examples of lines misclassified as natural language text

Artifact	Origin
Conflict: multiple assets emit to the same filename 75002edf.chunk.js	JS
ArrayUtils.sol:34:32: TypeError: Invalid type for argument in function call. Invalid implicit conversion from function (uint256) pure returns (uint256) to function (uint256) pure external returns (uint256) requested.	C++
Cannot determine embedded database driver class for database type NONE. If you want an embedded database please put a supported one on the classpath.	Java
Checking for update of app ”activity” in appstore	PHP
You must be using the interactive console to authenticate	PHP
- Theming: 1.12.0	PHP
Matplotlib: 1.4.3	Python
http://www.pcl-users.org/3rd-party-include- file-in-pcl-recognition-missing-if-pkg- config-not-available-td4031656.html	CPP
0x00007ffff6252acf in ?? () from /usr/lib/pymodules/python2.6/numpy/core /umath.so	Python
DEBUG [main] - $\texttt {<==}$ Total: 1	Java

**RQ2: Are artifact detection models trained on one programming language transferable to other programming languages?** Along the lines of our experiment for RQ1, we keep the training set size fixed at $n=$ 200 000. Again we apply our approach and train each language model 100 times. In contrast to RQ1, we now evaluate the resulting models on all languages’ validation sets to measure model transferability.

**Fig. 7:**
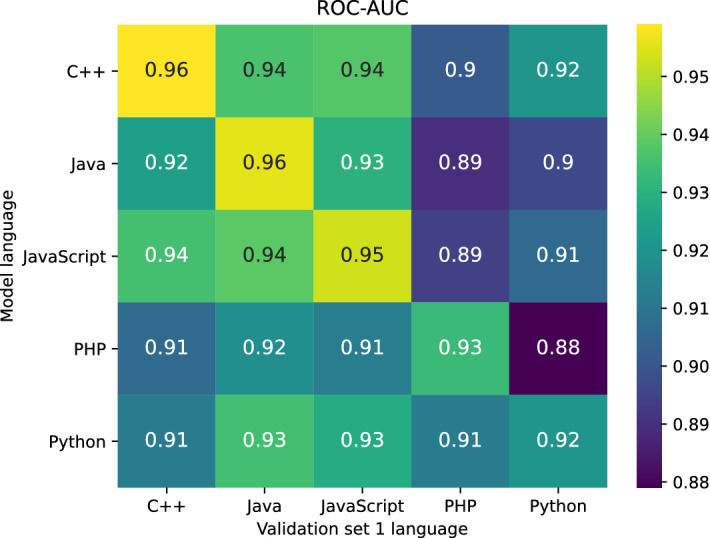
ROC-AUC matrix of models trained on a specific language ($n=$ 200 000, 100 repetitions) scored against each language specific Researcher 1 validation set

Figure 7 shows the mean ROC-AUC performance of models trained on a specific language performing predictions on the validation set of each language. Java, JavaScript, and C++ form a group that performs rather well when cross validated against each other. However, transfer of these models to PHP and Python is penalized by a significant drop in performance. Interestingly, while Python and PHP models do not start off with high performance on their respective language validation sets, their performance transfers well onto Java, JavaScript, and C++ validation sets.

**RQ3: What is the performance of a multi-language model for artifact detection?** We create a multi-language training set by taking equal sized and balanced samples from each language. Again, we keep the training set size fixed at $n=$ 200 000 to enable comparison to the models produced in RQ2, therefore containing 40 000 items from each of our five language specific data sets. Evaluation has been performed on Researcher 1 validation sets.

**Fig. 8:**
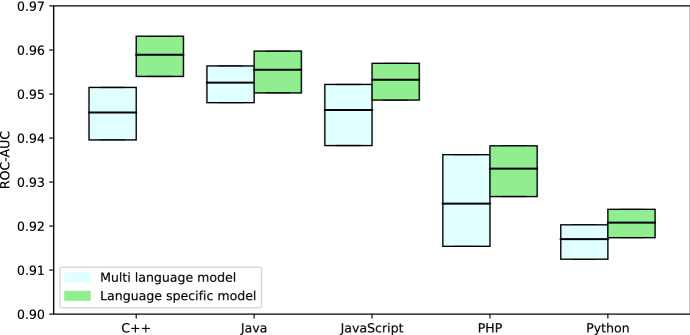
ROC-AUC bootstrap confidence interval ($\alpha =0.95$, n = 200 000, 100 repetitions) comparing the multi-language model to language specific models on the language specific Researcher 1 validation sets

Figure 8 shows the bootstrap confidence intervals and means of the multi-language models’ performance compared to the language specific models’ performance on their respective languages. The language specific models’ performance on its own language validation set is statistically significant better than the multi-language models’ performance (Student’s T-test: C++ $p=5*10^{-82}$, Java $p=10^{-17}$, JavaScript $p=2*10^{-35}$, PHP $p=3*10^{-30}$, Python $p=5*10^{-33}$).

Table 10 shows the performance of our models average over all languages validations sets. While the ROC-AUC performance scores of models evaluated on a specific language validation set are normal distributed, this is no longer the case when accumulating all validation sets’ scores. We therefore use one-sided Wilcoxon signed-rank test to compare the single language models accumulated scores again those of our multi-language models. The null hypothesis being that the single language model performs better or equal to the multi-language model, the corresponding p-values are shown in Table 10 for each model.

While the language specific models perform better on their specific language than the multi-language model (see Figure 8), the multi-language model performs better on average over all languages validation sets than any of the single language models (see Table 10). The implications for practical use of our models are: If the target dataset is sourced from projects with the same programming language, a single language model for this specific language is best. However, if the target dataset is spanning multiple programming languages, it may be unfeasible to use multiple single language models and separate the dataset to apply them accordingly. In such a scenario a multi-language model will perform significantly better than any standalone single language model.

**Table 10:** Model performance averaged over all languages Researcher 1 validation sets, p-values for null hypothesis that the model performs better than the multi-language model (Wilcoxon signed-rank test)

Model language	Mean ROC-AUC	*p*
C++	0.93	$2*10^{-18}$
Java	0.92	$10^{-11}$
JavaScript	0.93	$7*10^{-18}$
PHP	0.91	$10^{-15}$
Python	0.92	$10^{-16}$
Multi-language	0.94	

We conclude that our multi-language model outperforms all single language models when confronted with documents from multiple languages. However, given only a single target language, the language specific models outperform our multi-language model.

### Threats to validity

The biggest threat to internal validity are the static validation sets. While the training sets are repeatedly randomly sampled, the validation sets remain the same. We counteract this threat by creating rather big validation sets from 250 issue tickets for each of our target languages, with resulting sizes ranging between 5 000 and 10 000 lines per language. Further, manually labeling the validation sets is subject to human error, and also subject to human preference regarding what is actually considered an artifact or natural language. Therefore, two researchers independently classified the bug reports for the validation sets and we computed the inter-rater agreements on the validation sets to serve as indicator for dataset quality.

The biggest threat to external validity is the generalizability to other programming languages. While we conducted experiments with issue tickets of projects written in five different programming languages, the results might not be transferable to other programming languages. However, during the manual labeling of the validation sets, we observed that many similar artifacts are contained in issue tickets of all programming languages, e.g., html, xml and json snippets.

Furthermore, we used in our experiments only issue tickets from open source projects that are hosted on GitHub. Therefore, we cannot generalize our results to closed source projects and to projects hosted on other platforms where there might be other practices and habits for reporting and formatting issue tickets.

Another threat to the external validity is restriction to English projects where the majority of the issue tickets are written in English. It is up to future work to evaluate the performance of our approach on projects and issue tickets addressing different languages.

## Conclusion

We investigated the application of ML models to distinguish natural language portions of issue tickets from non-natural language artifacts. Our approach is comprised of an automated training set creation process, a custom preprocessing pipeline for the task, and a supervised ML model. In our previously published work (Hirsch and Hofer 2021), we performed this task on a dataset created from Java open source projects and compared our approach to NLoN (Mäntylä et al. 2018). In this work we focused on the portability and transferability of our models on five datasets spanning five different programming languages, based on our manually annotated validation sets. Our approach works best on data from C++, Java, and JavaScript, and moderate for PHP and Python data sets because artifacts in PHP and Python more often than in other languages, either closely resemble, or constitute proper English language sentences.

In practical application such a classifier may be used on data from projects spanning multiple programming languages, we therefore cross evaluated our models on documents for other programming languages. We observed that C++, Java, and JavaScript models performed well on each others’ documents, while dropping in performance when used on documents from PHP and Python, and vice versa.

As none of these single language models transferred well to all five programming languages, we investigated the applicability of multi-language models. Single language models slightly out-performed the multi-language models when evaluated on their native programming language. However, the average performance of multi-language models over all languages was significantly better than any of the single language models for the same use case. Based on the data that is to be processed, the user has to decide whether a single language model or multi-language model is better suited for the task.

Our models are intended to be used for preprocessing issue tickets and bug tickets, to remove noise that may negatively affect downstream NLP and IR applications. The supplied models’ classification performance is high, they are fast in prediction, while at the same time having a rather small memory footprint. However, the main advantage of our models arises from our automated training set creation process: the application of our models requires only little manual labor and effort, compared to the labor-intensive creation of regular expressions for the same task. While some Markdown annotated bug reports are required for training, the resulting models are agnostic to Markdown annotations and can classify inputs lacking such annotations.

The application of NLP and IR approaches on textual bug reports and issue tickets is an active research field. These methods are applied to solve a wide variety of tasks, for example, classification (Zhou et al. 2021; Thung et al. 2012; Chawla and Singh 2015), automated assignment (Mani et al. 2019; Devaiya et al. 2021), and fault localization (Zhou et al. 2012; Saha et al. 2013; Ye et al. 2016). Some approaches require non-natural artifacts to be removed or treated separately, for example, language identification (Jauhiainen et al. 2017), bug type classification (Hirsch and Hofer 2022a), and personality analysis (Calefato et al. 2019). This work intends to provide an alternative to labor intensive creation of regex for the task of artifact removal. Our models are implemented in Python 3, and we made all data, models, and implementations publicly available, including pretrained models ready to be used as a preprocessor for downstream tasks. We hope that this work and the resulting implementation and models can help other researchers in their work on textual bug reports.

In future work, we will investigate the application of our noise removal approach in practice and the resulting performance implications on a number of existing NLP and IR tasks, for example, language detection, automated ODC classification, root cause classification, and IR based fault localization. We will extend our approach to multi-class classification, in order to allow identification of specific types of artifacts as required for some tasks (e.g., stack traces for IR based fault localization). We will further expand our evaluations onto more datasets from various programming languages and other markup languages and data formats used in bug trackers.

## Data Availability

All accompanying datasets and implementations, including processed analysis targets and result data, are made publicly available on Zenodo (Hirsch and Hofer 2022b). Further, all implementations and results are also made available on GitHub. (https://github.com/AmadeusBugProject/artifact_detection/releases/tag/v1.2)

## Footnotes

1 see e.g. https://github.com/redisson/redisson/issues/2291 with 200 kB of uncompressed text

2 see e.g. https://github.com/haraldk/TwelveMonkeys/issues/37

3 see Stackoverflow 2021 Developer Survey https://insights.stackoverflow.com/survey/2021#most-popular-technologies-language

4 https://github.com/bazelbuild/bazel/issues/3906

5 see e.g. https://github.com/nextcloud/server/issues/16415

6 https://guides.github.com/features/mastering-markdown/

7 We evaluate Markdown usage in our mined dataset in Sect. 5.3.

8 see e.g. http://github.com/realm/realm-java/issues/3728

9 see e.g. http://github.com/realm/realm-java/issues/3047

10 see e.g. https://github.com/ReactiveX/RxJava/issues/6390

11 see e.g. http://github.com/dbeaver/dbeaver/issues/5156

12 https://insights.stackoverflow.com/survey/2021#most-popular-technologies-language-prof

13 https://github.com/topics/java?l=java &o=desc &s=stars, https://github.com/topics/cpp?l=cpp &o=desc &s=stars, https://github.com/topics/python?l=python &o=desc &s=stars, https://github.com/topics/php?l=php &o=desc &s=stars, https://github.com/topics/javascript?l=javascript &o=desc &s=stars, all accessed in February 2022

14 We had to ignore the documentation dataset of MuseScore (https://github.com/musescore/MuseScore) as this project repurposes the *.md* file ending for MuseData import files.

15 If the underlying data set was smaller than the desired training set size, the full data set size was used for this final evaluation. This was the case for the C++ training set.

16 https://github.com/dbeaver/dbeaver/issues/5156

17 https://github.com/pandas-dev/pandas/issues/14262
